# The Medical Witness

**Published:** 1899-10

**Authors:** 


### *EDITORIAL NOTES AND COMMENTS.*

The Business office of THE JOURNAL-RECORD is 305, 306 Fitten Building.

The Editorial office is 318-319-320 The Prudential.

Address all Business communications to Dr. M. B. Hutchins, Mgr.

Make remittances payable to THE ATLANTA JOURNAL-RECORD OF MEDICINE.

On matters pertaining to the Editorial and Original Communications address Dr. Bernard Wolff, Atlanta.

Reprints of original articles will be furnished at cost price. Requests for the same should always be made *on the manuscript.*

We will present, post-paid, on request, to each contributor of an original article, twenty (20) marked copies of THE JOURNAL-RECORD containing such article.

### THE MEDICAL WITNESS.

ALL physicians in the State, and certainly those who belong to the State Medical Association, have in the last few weeks received the following notice:

ATLANTA, September 1, 1899.

DEAR DOCTOR:—The undersigned committee of the Medical Association of Georgia invites your special attention to the facts and the suggestions contained in the accompanying paper, as of direct and personal interest to every medical practitioner in the State.

Bills providing for the payment of expert medical witnesses and making confidential communications to physicians privileged communications will be carefully prepared and will be presented at the approaching meeting of the General Assembly in October next.

Please communicate promptly with the representatives from your county and with the State senator from your district in advocacy of these measures—urging the subject upon their attention and securing, if possible, their favorable consideration.

Be kind enough to convey the result of your interviews to the chairman of the committee at your earliest convenience and greatly oblige,

Yours very truly,

JAMES B. BAIRD, M.D., Atlanta, Chairman,
E. H. RICHARDSON, M.D., Atlanta,
F. M. RIDLEY, M.D., LaGrange,
GEORGE H. NOBLE, M.D., Atlanta,
L. G. HARDMAN, M.D., Harmony Grove,
W. S. ELKIN, M.D., Atlanta,
J. B. MORGAN, M.D., Augusta,
C. D. HURT, M.D., Atlanta,
J. L. WALKER, M.D., Waycross,
THOMAS R. WRIGHT, M.D., Augusta,
Committee on Medical Legislation.

Those of us who were present at the last meeting of the Association at Macon will remember the timely paper read by Dr. J. B. Baird on "The Medical Witness." In this paper he took the position that "the laborer is worthy of his hire," and that there are many things which are confidential between the patient and physician which no law should compel the latter to divulge. Physicians everywhere must recognize the justice of these two propositions. It is certainly an injustice to a physician who has spent thousands of dollars in acquiring this professional knowledge, to be compelled by the laws of the State to testify as an expert and yet receive only the fee, $2 per diem, of the ordinary witness. Dr. Baird has called attention to the fact that several States have statutes providing for a reasonable remuneration for expert services, among which are Iowa, New York, North Carolina, and Rhode Island. The other proposition is equally valued in regard to professional secrets, and several of the States have enacted laws to protect physicians against compulsory testimony. We trust every physician in the State will read carefully the purport of this circular sent out by the committee appointed by the State Medical Association. We deserve to have a law enacted which will protect a physician and his patient, and to protect his professional knowledge, which is his ware in trade. We can only accomplish this by united effort from every physician in the State. Make it a point to see your representative and senator personally and present before him the reasonable necessity for the passage of such a law. The physicians must stand together if we desire laws enacted for their benefit. Start at once, and as you progress let the chairman, Dr. Baird, know what you are doing. R.

